# Progress and Challenges in HIV-1 Vaccine Research: A Comprehensive Overview

**DOI:** 10.3390/vaccines13020148

**Published:** 2025-01-31

**Authors:** Alex C. Boomgarden, Chitra Upadhyay

**Affiliations:** Division of Infectious Diseases, Department of Medicine, Icahn School of Medicine at Mount Sinai, New York, NY 10029, USA; alex.boomgarden@mssm.edu

**Keywords:** HIV-1 vaccine, broadly neutralizing antibodies, signal peptide, germline-targeting, mRNA, adeno-associated virus

## Abstract

The development of an effective HIV-1 vaccine remains a formidable challenge in biomedical research. Despite significant advancements in our understanding of HIV biology and pathogenesis, progress has been impeded by factors such as the virus's genetic diversity, high mutation rates, and its ability to establish latent reservoirs. Recent innovative approaches, including mosaic vaccines and mRNA technology to induce broadly neutralizing antibodies, have shown promise. However, the efficacy of these vaccines has been modest, with the best results achieving approximately 30% effectiveness. Ongoing research emphasizes the necessity of a multifaceted strategy to overcome these obstacles and achieve a breakthrough in HIV-1 vaccine development. This review summarizes current approaches utilized to further understand HIV-1 biology and to create a global vaccine. We discuss the impact of these approaches on vaccine development for other diseases, including COVID-19, influenza, and Zika virus. Additionally, we highlight the specific limitations faced with each approach and present the methods researchers employ to overcome these challenges. These innovative techniques, which have demonstrated preclinical and clinical success, have advanced the field closer to the ultimate goal of developing a global HIV-1 vaccine. Leveraging these advancements will enable significant strides in combating HIV-1 and other infectious diseases, ultimately improving global health outcomes.

## 1. Introduction

HIV-1/AIDS remains one of the most significant global health challenges. Despite substantial progress in treatment and prevention, the epidemic continues to affect millions of people worldwide. As of 2023, approximately 39.9 million people globally were still living with HIV-1 [1]. While new infections have decreased by 60% since 1995, 1.3 million new cases were reported in 2023. Since the epidemic began, 88.4 million people have been infected, and 42.3 million have died from AIDS-related illnesses, including 630,000 deaths in 2023.

Antiretroviral therapy (ART) has significantly improved the quality of life and life expectancy for those living with HIV-1; however, patients are not cured [2,3]. ART utilizes the combination of multiple medications (typically 2 to 4) that target various stages of the HIV-1 life cycle, enabling patients to display negligible/undetectable levels of the virus and healthy CD4+ T cell levels. As a result, ART can also significantly reduce the transmission of HIV-1 between individuals. Despite its success and widespread availability, many people, particularly in low- and middle-income countries, face significant barriers to accessing ART [4]. Additionally, lifelong adherence to ART is required to manage HIV-1, and its toxicity can increase the risk of several comorbidities (i.e. cardiovascular disease, liver disease) in aging individuals [5]. This ongoing challenge underscores the need for a vaccine that can prevent transmission, reduce new cases, and ultimately control the spread of the virus to achieve global eradication. 

## 2. Historical Context

**Figure 1:**
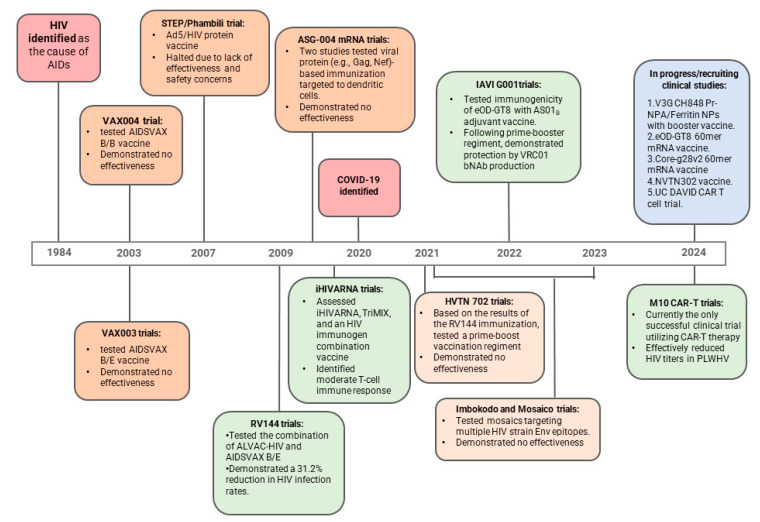
Historical context of HIV-1 research. Since its identification in 1984, HIV-1 has been the focus of decades of research aimed at developing a global vaccine. This timeline showcases the saga of clinical trials outcomes. A major turning point in the success of clinical trials arose after the RV144 trial, which provided not only vital data regarding HIV-1 vaccination but also hope for the arrival of a global vaccine in the near future. Several clinical trials are now in development or progress which utilize the recent advances in HIV-1 vaccine technology described in this review.

The quest for an HIV-1 vaccine has been a challenging journey marked by numerous setbacks (Figure 1). After HIV-1 was identified as the cause of AIDS in 1984, there was initial optimism by research and medical professionals that a vaccine could be developed rapidly. The first HIV-1 vaccine Phase I clinical trial began in 1987, but early candidates, such as the gp160 subunit vaccine, showed no significant efficacy [6,7,8]. 

In 1998, the first large-scale, randomized, double-blind, placebo-controlled HIV-1 vaccine trial (VAX004) was launched in the US and Europe. This Phase 3 trial tested the AIDSVAX B/B vaccine, which included two recombinant gp120 envelope (Env) proteins from the subtype B isolates MN and GNE8 [9]. Unfortunately, when the study concluded in 2003, it showed no effectiveness in reducing HIV-1 infection or plasma viremia levels. Another major Phase 3 trial, VAX003, was conducted in Thailand. This trial used the AIDSVAX B/E vaccine, which combined gp120 Env proteins from clade B (MN) and clade E (A244) [9]. Like VAX004, this trial also failed to demonstrate protection against HIV-1. The disappointing results from VAX004 and VAX003, both conducted by VaxGen, Inc., highlighted a crucial point: simply producing antibodies that can bind to the HIV-1 Env is not enough to protect against the virus or lower the viral load. 

Undeterred by these initial setbacks, in 2007, the STEP and Phambili trials tested a vaccine developed by Merck that used a modified adenovirus type 5 (Ad5) vector to deliver HIV-1 genes for three HIV-1 proteins: Gag, Pol, and Nef from subtype B [10,11]. The goal was to stimulate a strong cellular immune response, particularly from CD8+ T cells, which can kill HIV-1-infected cells, and CD4+ T cells, which can help activate other immune cells (e.g. antibody secreting B cells) [12,13]. Unfortunately, both trials were halted due to safety concerns and a lack of effectiveness. This experience underscored the complexity of developing an effective HIV-1 vaccine and highlighted the need for continued research and innovation.

The first glimmer of hope in the HIV-1 vaccine saga came with the RV144 trial, also known as the “Thai Trial”. This Phase 3 trial, conducted in Thailand, began in 2003 and involved over 16,000 volunteers. It tested a combination of two vaccines: ALVAC-HIV (a canarypox vector vaccine coding for HIV-1 env, gag, and pol) and AIDSVAX B/E (a gp120 subunit vaccine). The results, announced in 2009, were groundbreaking. The vaccine regimen demonstrated a modest 31.2% reduction in HIV-1 infection rates compared to the placebo group [14,15]. While this level of efficacy was not sufficient, it was the first time any HIV-1 vaccine had shown a protective effect in humans. The RV144 trial’s success, albeit limited, rekindled hope in the scientific community and provided crucial insights into the immune responses needed for protection [16,17,18,19,20,21,22]. In an effort to recapitulate the success of RV144, another trial was launched in South Africa, the HVTN 702 (Uhambo) Trial, which tested a modified version of the RV144 vaccine regimen. However, it was stopped early because interim results showed that it did not prevent HIV-1 infection [23,24]. Another set of trials, the Imbokodo and Mosaico Trials (2021–2023), tested vaccines developed by Janssen using mosaic immunogens designed to protect against multiple HIV-1 strains. Unfortunately, both trials were halted after failing to show efficacy [25]. Despite the setbacks over the decades, these trials have significantly advanced our understanding of HIV-1 to inform the experimental design of subsequent studies. Moreover, they have highlighted the need for innovative methodologies and technological advancements to achieve a breakthrough in the quest for an HIV-1 vaccine. These trials also established that binding Abs and cellular immunity may not suffice for protection. Rather, the elicitation of Abs that can neutralize HIV-1 isolates from diverse clades, termed broadly neutralizing Abs (bNAbs), will be needed to offer protection. In upcoming sections, we will describe initiated or completed studies utilizing various new vaccine technologies, which have shown significant improvements following the COVID-19 pandemic. 

## 3. Why Is It Difficult to Develop an HIV-1 Vaccine?

Developing an HIV-1 vaccine has been exceptionally challenging for several reasons. The primary target for HIV-1 vaccine development is the Env glycoprotein, the only viral protein exposed on the surface of the virus, infected cells, and to the host environment, making it the sole target for host antibodies. The Env protein is initially synthesized as a gp160 precursor, which is then cleaved into gp120 and gp41 subunits. The gp120 subunits contain the receptor (CD4) and coreceptor-binding sites (CCR5 and CXCR4), while gp41 contains the fusion machinery. Together, three gp120 and three gp41 subunits form a trimeric Env spike. While these characteristics are comparable to other viruses such as SARS-CoV (spike) and influenza (hemagglutinin), which also have trimeric surface proteins, HIV-1 presents unique challenges in the pursuit of a vaccine. Some of the key challenges are discussed below.

One major obstacle in developing an HIV-1 vaccine is the extraordinary genetic diversity of HIV-1, with multiple subtypes and strains circulating globally [26]. This diversity arises from the virus’s rapid mutation rate (i.e. 3.4 × 10^-5^ mutations per base in one replication cycle), particularly in the Env protein, due to the lack of proof reading by reverse transcriptase. This results in millions of Env variants in an infected individual within 24 h [27]. These Env variants can exhibit significant molecular and structural alterations, allowing the virus to evade host-mounted antibodies. This makes it exceedingly difficult to create a vaccine that can provide broad protection across all variants [28]. While people living with HIV (PLWH) naturally develop bNAbs that can neutralize various HIV-1 strains, inducing these antibodies through vaccination has proven extremely challenging. These bNAbs evolve only after the extensive and prolonged somatic hypermutation (SHM) of precursor antibodies [29,30,31]. Additionally, the low density of Env proteins (~7–14 spikes per virions) on the virus and their unique properties make them poor immunogens, complicating the induction of bNAbs through vaccination [32,33,34]. 

The relatively conserved regions of the HIV-1 Env protein, which could be targeted by antibodies, are shrouded by a dense layer of glycans, known as the “glycan shield”. This shield, comprising about 50% of gp120’s mass, acts as a protective layer masking the conserved neutralizing epitopes on the Env [35,36]. Other vulnerable sites on the Env protein are only transiently exposed during receptor-triggered conformational changes that facilitate the fusion of the virus with host cell membranes [37,38,39]. This structural protection, combined with the Env protein’s ability to tolerate mutations without losing functionality, allows HIV-1 to rapidly escape from naturally developed or therapeutically administered bNAbs [37,40,41,42]. Additionally, the ability of the virus to establish latent viral reservoirs makes it challenging to eradicate the virus. These reservoirs typically comprise HIV-1-infected CD4+ T cells, which can remain dormant for years without producing viral particles thereby evading the immune system and ART [43]. While specific gene therapies and drugs developed have been tested to target these reservoirs by activating latent HIV-1-infected cells (i.e. shock-and-kill approach), these methods have had limited clinical success [44,45,46]. 

Another obstacle is the incomplete knowledge of correlates of protection, largely due to the delay in a successful clinical trial to glean information from, which we did not see until the RV144 study. Moreover, HIV-1 infected individuals can produce antibodies or exhibit CD4 T cell activation without developing immunity, making it difficult to establish physiological benchmarks for protection. Another caveat is that for the immune system to produce a primary antibody response or a cytotoxic T cell response, it needs the help of CD4 T cells. However, in the case of HIV-1, the virus tends to down regulate CD4 on CD4 T cells, and we do not have reliable markers to measure the support provided by CD4 T cells. While we can identify markers that show that CD4 T cells are activated, these markers do not necessarily predict how effective the immune response will be. Current studies suggest that correlates to immunity involve a complex combination of several immune responses [47,48]. While eliciting bNAbs via vaccination is the goal, non-neutralizing antibodies (nNAbs) cannot be ignored. The partial success of RV144 trial highlighted the role of nNAbs in HIV-1 protection [49]. Both bNAbs and nNAbs can reduce the plasma viral load via Fc-mediated functions in humanized (hu)-mice and non-human primates (NHP) models [50,51,52,53,54,55,56,57,58,59,60]. Similar findings are reported with nNAbs against West Nile and influenza viruses [61,62,63,64]. Thus, nNAbs with Fc functions can confer protection or alter the course of HIV-1 infection by binding to cell-free virions to accelerate virus clearance, and/or by binding to HIV-1 Env on the surface of infected cells to induce antibody-dependent cell-mediated cytotoxicity (ADCC) and monocycle/macrophage phagocytosis [57,65,66,67]. Thus, a successful vaccine would need to engage both the innate cellular and humoral arm of the immune response, as antibody production or CD4 T cell activation alone have had limited success in controlling HIV-1 infections. 

Here, we will discuss the ongoing research, technological advancements, and innovative approaches that bring renewed hope for an effective HIV-1 vaccine to be developed in the near future.

## 4. Recent Technological Advances

A significant breakthrough in vaccine design has been the identification of highly potent bNAbs against HIV-1. Since the first bNAb (b12) was identified in the early 1990s [68], several more potent bNAbs have been isolated, including PG9 and PG16, which neutralize approximately 70-80% of HIV-1 strains, VRC01, with a neutralization breadth of around 90%, and 10E8, which neutralizes 98% of tested viruses [69,70,71]. All bNAbs, or so called ‘super-antibodies’ target conserved epitopes on the trimeric Env on virions that remain relatively unchanged despite its high mutation rate. In addition, these bNAbs also possess unique features such as extensive SHM and/or a long complementarity-determining region (CDR) [72,73]. The isolation of exceptionally broad and potent bNAbs has facilitated the identification of five key targets on the HIV-1 Env: (1) V2 site at the trimer apex, (2) N332 supersite, (3) CD4 binding site (CD4bs), (4) gp120–gp41 interface, and (5) membrane-proximal external region (MPER). Proof of concept passive transfer studies in hu-mice and NHP have shown that the administration of high doses of bNAbs can afford sterilizing protection demonstrating the potential of virus-specific humoral immunity [42,74,75,76,77,78,79,80,81]. While the direct administration of bNAbs has limitations (e.g. viral diversity and resistance), their study has established a consensus that immunogens targeting these specific sites on HIV-1 Env can enhance the potential for developing broadly effective vaccines that can contribute to better control and global prevention of HIV-1 infection. This understanding has driven the development of the SOSIP trimer, a mimic of the HIV-1 Env, to better present these critical sites to the host immune system.

### 4.1. Immunization with SOSIP Trimers

The design and development of SOSIP trimers marks a significant advancement in HIV-1 vaccine research [82,83]. SOSIP trimers mimic the native structure of the HIV-1 Env trimer, which is deemed crucial for eliciting bNAbs. Using computational and structure-guided design, multiple stabilizing mutations were incorporated to improve the stability and to lock the trimer in a closed, native-like state. This includes linking gp120 and gp41 subunits with a covalent disulfide bond (SOS) and stabilizing the pre-fusion form of gp41 with an I559P point mutation (IP) [82,83,84,85]. Several studies have shown that the recombinant SOSIP trimer maintains stability and conformational integrity compared to previously studied Env proteins [86].

Preclinical studies have shown that SOSIP trimers can induce potent neutralizing antibody responses in animal models, although these responses are often strain-specific. To address this, researchers are exploring multivalent vaccine strategies that incorporate SOSIP trimers from multiple HIV-1 strains to broaden the immune response. The SOSIP platform has also been adapted to modifications, such as the removal/filling of glycan holes and the optimization of glycan occupancy, to improve the presentation of Env to the immune system and enhance the breadth and potency of elicited antibodies. Initially, isolate BG505 was used, later expanding to multiple strains from different clades to address the challenges associated with the enormous sequence diversity of HIV-1 [26,82,85,86,87,88,89,90,91,92,93,94,95,96,97]. Immunizing rabbits with the soluble, cleaved, trimeric form of HIV-1 (JR-FL_SOSIP) using a DNA prime and protein boost strategy produced antibodies that neutralized sensitive HIV strains at high titers. However, the breadth against heterologous primary isolates was limited [98,99]. The immunization of conventional mice (i.e. BALB/cJ, C57BL/6J, and 129S1/SvImj) with soluble BG505 SOSIP.664 trimers elicited Env-binding IgG antibodies and robust T follicular helper (Tfh) cell and germinal center (GC) responses, but did not produce BG505.T332N neutralizing antibodies [100]. Epitope mapping revealed that non-neutralizing epitopes on the base of soluble Env are not well shielded, unlike on virions, prompting the further refinement and modification of the SOSIP trimers [92,101]. Recently, Wang et al presented findings from a Phase I clinical trial (VRC 018) demonstrating that DS-SOSIP, a BG505 Env trimer, stimulated the production of bNAbs N751-2C06.01 and N751-2C09 in mice [102]. Given this success, SOSIPs eliciting the production of these antibodies are likely to be key immunogens for future clinical studies. This has prompted researchers to utilize SOSIP trimers in combination with various other methods of vaccine delivery, including mRNA and nanoparticle technology, to maximize efficacy, cost, and accessibility. These studies, which have demonstrated tremendous success, are discussed in subsequent sections. 

While SOSIP trimers hold great potential for HIV-1 vaccine development, several challenges remain. Among these is the use of adjuvant formulations. In animal studies, the low pH or DNA components of specific adjuvant formulations have the capacity to destabilize SOSIP trimers, thereby impeding proper immunogenicity [103]. These findings indicate the necessity for further research to determine highly efficacious combinations of adjuvants with a given vaccine. Furthermore, the preclinical and clinical studies involving SOSIP trimers largely contain a non-native signal peptide (SP) coding sequence, particularly the SP from tissue plasminogen activator (tPA). Mounting evidence suggests that the native SP of HIV-1 Env is crucial for Env folding, glycosylation, and the overall presentation of the Env, beyond merely targeting the protein to endoplasmic reticulum (ER) [104,105,106]. Studies have shown that using heterologous SP even from other HIV-1 isolates can affect overall Env properties, including glycan occupancy, composition, and the neutralization sensitivity of the virus to antibodies. Recent studies indicate that SP can regulate the capacity of Env to elicit binding and functional antibodies. The immunization of mice with Env expressed with native wild-type SP elicited weak tier 2 neutralizing antibodies, which were diminished upon swapping the SP [107]. It will be interesting to compare the influence of tPA SP and HIV-1 native SP on different Env properties, including immunogenicity. As we previously introduced and touched upon briefly, another notable limitation of SOSIPs is the exposure of the gp41 trimer base, which is typically masked by the viral membrane [95,108]. This has likely led to confounding data reporting off-target bNAbs directed to these sites. This limitation has been taken into consideration by many researchers; among those, some have chosen to introduce N-linked glycans to cover these exposed surfaces [109]. Another limitation of SOSIPs, which is highly debated amongst researchers, is that the SOS bond itself may introduce non-native, structural changes. Supported by single-molecule fluorescence resonance energy transfer (smFRET) studies, SOSIPs appear to favor downstream conformations (states 2 and 3) compared to a closed conformation (state 1) [110,111]. Research by Zhang et al indicates that swapping the SOS modification with an interdomain lock (IDL) within gp120 stabilizes Env in a more native, state 1 conformation [108]. 

Overall, the SOSIP trimer design is a pivotal development in the quest for an effective HIV-1 vaccine, providing a robust platform for presenting the Env spike in a form that closely resembles its native structure on the virus. However, despite its potential, SOSIP trimers have not been successful at eliciting cross-clade neutralizing antibodies consistently. This underscores the need for further refinement and optimization to improve their immunogenicity and potency in eliciting a bNAb response. 

### 4.2. Germline-Targeting (GT) Immunization

Perhaps one of the more exciting new approaches and largely reliant on the use of SOSIPs is the development of germline-targeting (GT) immunization. When considering PLWH, a fraction of these individuals develop bNAbs over long periods of time due to the co-evolution of the virus/Env and humoral responses [69,112,113,114,115,116,117,118,119,120]. This humoral response involves the conditioning of B cells to undergo SHM in the germinal center (GC), which facilitate the parallel development of bNAbs effectively targeting the wide spectrum of Env constituting a given HIV-1 infection. This understanding led researchers to attempt to artificially recreate this co-evolutionary process in vitro to determine whether a similar process of generating highly effective bNAbs could be propagated by B cells [121,122]. 

In broad terms, successful GT immunization requires several rounds of immunogens, which both facilitate B-cell recruitment to GC followed by subsequent immunogen boosters to “teach” the B-cells to produce bNAbs. However, the characterization of the human IgG repertoire has revealed that the naive B cell precursors required to elicit bNAbs are extremely rare [123]. Nonetheless, recent advancements in GT immunization strategies have demonstrated significant progress in the field of HIV vaccine development [124,125]. 

In 2024, multiple groundbreaking preclinical studies were published demonstrating the tremendous capabilities of this technique. Ray et al. showcased the production of bNAbs from naïve B cells by targeting the membrane-proximal external region (MPER) epitope on gp41 subunits [126]. Published in the same issue of *Nature Immunology*, Schiffner et al utilized scaffold nanoparticles to stimulate MPER-targeted bNAbs in mice B-cells [127]. Additionally, Caniels et al demonstrated that BG505 SOSIP germline trimer 1.1 (GT1.1) immunogen, can engage a variety of VRC01-class bNAb precursors. In knock-in mice, a single dose of GT1.1 expands CD4bs specific VRC01-class B cells and drives their maturation [128], while a similar approach in NHPs led to the identification of bNAbs 12C11 and 21N13s, which effectively neutralized a subset of diverse, heterologous, neutralization-resistant viruses. These findings have garnered significant attention, as the GT1.1 SOSIP trimer is currently the focus of a Phase 1 clinical trial. 

Furthermore, Wang et al. reported the success of GT immunization using an engineered outer domain germline targeting version 8 (eOD-GT8) 60mer, a soluble self-assembling nanoparticle, delivered via lipid nanoparticle–encapsulated nucleoside mRNA (mRNA-LNP). This facilitated the production of VRC01-like bNAbs from B-cells in mice [129]. Clinical studies have been completed utilizing the concepts of GT to test its potential as a vaccine in human subjects. In 2022, findings from the IAVI G001 Phase 1 clinical trial showed that 97% of participants who received the eOD-GT8 vaccine developed VRC01-class IgG B cell precursors [130,131]. This trial was the first stage in a multi-stage HIV-1 vaccine regimen, and further boosting these responses is expected to drive these precursors towards the maturation pathway. Nonetheless, these results were very encouraging and represent a significant step forward in the development of an effective HIV-1 vaccine [130,131]. Building off these findings, it was determined that two vaccinations of eOD-GT8 60mer induced a strong response from antigen-specific CD4+ T cells [132]. While the use of the eOD-GT8 60mer also represents an mRNA- and nanoparticle-based vaccine, a more in-depth description of these forms of immunization is described below.

Despite the enormous potential of GT immunization for developing an HIV-1 vaccine, it is a highly complex cellular process with unique challenges. One major challenge is the requirement to teach the B cells to produce not one but a pool of bNAbs that provide protection. To address this, researchers have utilized resources such as ultradeep antibody sequence databases to identify pools of bNAb precursors with shared structural features capable of binding to conserved HIV Env sites. This structure-guided design has led to the development of highly effective HIV Env immunogens capable of stimulating B-cell bNAb precursor production in mouse and NHP models [133,134,135]. Similar methods used in future GT immunization studies can provide valuable insights for developing an HIV-1 vaccine. 

### 4.3. mRNA-Based Vaccines

Messenger RNA (mRNA) vaccines, considered newcomers in the field, represent a groundbreaking advancement in immunology. This innovation led researchers, who pioneered this work, Katalin Kariko and Drew Weissman to win the 2023 Nobel Prize in Physiology or Medicine [136]. Unlike traditional vaccines, which often use weakened or inactivated viruses to stimulate an immune response, mRNA vaccines use a small piece of the virus’s genetic material. This mRNA instructs cells to produce a protein that triggers an immune response, priming the body to recognize and fight the pathogen if encountered [137].

The application of mRNA technology to HIV-1 vaccine development is particularly promising due to several key factors: (1) Rapid development and adaptability: mRNA vaccines can be developed and produced much faster than traditional vaccines. This speed is crucial for responding to the diverse and rapidly mutating HIV-1 virus. Researchers can quickly update the mRNA sequence to match evolving HIV-1 strains, potentially improving vaccine efficacy while maintaining safety. (2) Strong immune response: mRNA vaccines have shown the ability to elicit strong cellular and humoral immune responses by the production of antigens directly from cells compared to protein/peptide vaccines. For HIV-1, this means the vaccine can stimulate the production of antibodies and immune cell activity to target the virus. Early-stage clinical trials of mRNA HIV-1 vaccines (described below) have demonstrated promising immune responses, including cytotoxic T cell activity, paving the way for further research. (3) Elicits bNAbs: One of the goals in HIV-1 vaccine development is to induce bNAbs that can neutralize a wide range of HIV-1 strains. mRNA vaccines can be designed to generate and present specific HIV-1 proteins that are known to elicit bNAb production, potentially offering broad protection against the virus [138,139,140]. Preclinical studies highlight the strengths of utilizing an mRNA-based approach to elicit an immune response. A common strategy involves the coupling of mRNAs with nanoparticle technology, which serves as both a delivery vehicle and protective shield from RNases [141,142]. This includes a recent study which cleverly packaged lipid nanoparticles (LNPs) with mRNAs targeting HIV-1 viral protease cleavage sites (VPCSs) at multiple sites. While this in vivo study reported no change in CD4+ T cell activation, they identified an increase in VPCS CD8+ memory T cells [143]. As CD8+ T cells’ “memory” can enhance their overall ability to recognize and kill HIV-1-infected cells, methods such as these are key in the design of an effective vaccine [12]. A separate study demonstrated a virus-like particle (VLP), displaying mRNA encoding Env and Gag proteins induce the production of bNAbs in mice [144]. These findings were particularly relevant, as they demonstrated that the use of both Env and Gag mRNAs stimulated a greater bNAb response compared to Env alone. Ahmed et al, also showed that mRNA-LNPs encoding HIV-1_AD8_ Env markedly enhanced neutralizing antibody production in rabbits [145].

Currently there are several completed and ongoing clinical mRNA-based HIV-1-vaccine candidates worth noting [138]. Among those completed are two ASG-004 mRNA studies, NCT00672191 and NCT00833781, which involved the transfection of dendritic cells with viral proteins (e.g. Gag, Nef). Unfortunately, both attempts at targeting DCs for HIV-1 immunization failed to stimulate an antiviral response [146,147]. Challenges in producing positive results from mRNA-based clinical studies continued with the HIVACAT-TriMIX mRNA study (NCT02888756), which was terminated due to the lack of immunogenicity identified at interim analysis [148]. Shortly thereafter, a Phase I dose-escalating clinical trial using a naked mRNA (iHIVARNA), TriMix (CD40L + CD79 + caTLR4 RNA), and a novel HIV-1 immunogen sequence (encoding Gag, Pol, Vif, and Nef) was completed. Unfortunately, their findings indicated only a moderate T cell immune response from vaccinated individuals [149]. Despite these setbacks, the motivation and confidence in this technology, instilled by the remarkable success of the COVID-19 vaccine, remain strong. As a result, several clinical trials have been developed and are currently recruiting candidates. These include (1) a Phase 1 clinical trial (NCT05903339) involving priming (V3G CH848 Pr-NP1; ferritin NPs expressing Env trimer) and booster (V3G CH848 mRNA-Tr2; mRNA-LNP) immunogens [139]; (2) eOD-GT8 60mer mRNA (NCT05414786) [130], (3) Core-g28v2 60mer mRNA (NCT05001373) [139], and (4) NIAID sponsored study HVTN 302, which is examining three HIV-1 mRNA vaccines coding for BG505 MD39.3, BG505 MD39.3 gp151, and BG505 MD39.3. As these studies represent many unique approaches to HIV-1 vaccination utilizing mRNAs, the research community eagerly awaits their results.

The success of mRNA vaccines against COVID-19 has not only influenced current/future HIV-1 vaccine design but has opened the door for their application to combat other diseases. The CDC estimated that seasonal influenza (flu) killed between 12,000 and 52,000 individuals between 2010 and 2020, highlighting the need for a universal influenza vaccine. This is in large part due to traditional flu vaccines showing variable effectiveness, typically between 40% and 60% [150], while mRNA vaccines offer higher efficacy by precisely targeting the most prevalent flu strains each season. Clinical trials are currently underway to compare mRNA flu vaccines, which have included the use of hemagglutinin (HA) to induce the production of bNAbs. In 2024, a Phase1/2 randomized clinical trial of the mRNA-1010 vaccine reported that a single dose can elicit higher hemagglutination inhibition (HAI) titers and T cell responses against influenza A strains [151,152]. These findings not only endorse their use and application for future flu vaccines but validate the effectiveness of mRNA-based vaccines to be used against other pathologies. mRNA vaccines are also being developed for Zika virus, with preclinical and clinical trials demonstrating strong immune responses against Zika [153,154,155]. A 2023 study showed that mRNA-1893 vaccines in adults induced the robust production of neutralizing antibodies against Zika [152]. Although the global impact of rabies virus (RABV) is smaller compared to the flu or Zika, it is nearly always fatal once symptoms appear, making an effective vaccine crucial. Preclinical studies in different animal models, including dogs, mice, and NHPs have shown that mRNA vaccines can elicit immunity [156,157,158]. As humans are often exposed to RABV through bites and scratches from animals, clinical trials in humans have been initiated to provide immunity and improve post-exposure prophylaxis. The anticipated or established success of these studies underscore the challenging nature of HIV-1 as a pathogen compared to others and highlight the complex road ahead for HIV-1 vaccine development.

While mRNA vaccines hold great promise, their use comes with a unique set of challenges. Among these is the difficulty in maintaining mRNA stability and storage. mRNA is inherently unstable and requires ultra-cold storage (i.e., between −130°F and −76°F) [159], posing logistical challenges, especially in low-resource settings. Advances in formulation and delivery methods are needed to improve the stability and ease of distribution [160,161]. Several strategies have been implemented to address stability and storage issues, including the following: (1) mRNA design and production methods: specific mRNA designs such as replacing the nucleotide uridine with pseudouridine can increase stability by reducing RNase-based degradation [162,163,164]. Alternatively, uridine depletion or increasing the overall GC content can have a similar effect on stabilization [162,165]. (2) Freeze-dried mRNA: the lyophilization of mRNA can enhance stability without jeopardizing immunogenicity. This has been shown particularly in reports involving mRNA-LNPs to maintain their efficacy following lyophilization [164,166,167]; (3) Kit-assembly approaches: representing an ‘at point of use’ approach, a clinical study (NCT0241077) demonstrated the efficacy of an mRNA-based cancer therapy which combined mRNA and cationic liposomes. This method has many advantages for future vaccines for pathogens such as HIV-1, by which components are stored separately to increase shelf life. Furthermore, the separation of components enables mixtures to be both individualized and combined upon request [168]. (4) The selection of pharmaceutical excipients: the composition and purity of excipients can enhance the stability of mRNA. Collectively, these strategies have improved the stability and by virtue, efficacy of mRNA-based vaccines including but not limited to HIV-1 [169]. 

A second challenge for mRNA vaccines is ensuring long-lasting immunity. One approach that researchers have found effective is booster doses and combination strategies, which has shown in recent studies to prolong the immune response. The success of mRNA-based COVID-19 boosters has strongly influenced their use in HIV-1 studies [170]. As mentioned previously, GT immunizations have reported positive results by which strong bNAb production can be stimulated. Building off these reports, researchers demonstrated that N332-GT5 membrane-anchored mRNA priming and boosting immunizations triggered long-lasting GCs and a prolonged humoral response in mice [171]. To mitigate the spread of a given pathogen, immunization among all classes of citizens is essential. This highlights a third and pivotal obstacle mRNA vaccines face, which is ensuring their accessibility to middle or low-income populations. While an HIV-1 vaccine is still unavailable, epidemiological studies focused on other pathologies and mRNA-based vaccines have revealed daunting disparities. A study conducted in the US using the 2021 Medical Expenditure Panel Survey (MEPS) indicated that lower income groups were 55% less-likely to be vaccinated for COVID-19 [172]. Moreover, a global survey examining COVID-19 vaccinations between 2022 and 2023 determined that a staggering 50% had an unmet demand for immunization [173]. Accessibility appeared lowest in African countries and highest in Western Pacific countries. Efforts are underway to scale up production and reduce costs to ensure global access. This has included the development of the International Treaty for Pandemic Prevention, Preparedness, and Response, which represents a new effort by global leaders to combat future pathogens by increasing vaccination accessibility [174]. It is important to note that recent surveys conducted in the US have indicated a significant rise in “vaccine hesitancy”, particularly following the COVID-19 pandemic [175,176]. This has placed heightened emphasis on health officials and fact checkers to combat the spread of misinformation to enhance vaccination rate.

Despite these clear obstacles, it is noteworthy to discuss the significant reduction in cost offered by mRNA vaccines. The concept of an mRNA-based vaccine gained momentum particularly in 1990s upon which researchers determined that in vitro transcribed mRNA could drive the production of proteins in eukaryotic cells and mouse tissue [137,177,178]. However, their development was hamstrung by skeptics raising concerns of their effectiveness and stability. Following their success in recent years, mRNA is now at the forefront of vaccine design to provide safe immunity against various pathogens all while reducing the cost of production for companies and patients. The reduced cost of mRNAs can first be attributed to their production in a cell-free system. Alternative methods, including protein subunits or viral vector vaccines, require the use of cell cultures and bioreactors, which can greatly increase costs. mRNAs can be produced using cell-free transcription systems with a single DNA template, which can be dramatically scaled up to increase production and reduce turnaround time. Moreover, mRNAs can encode for multiple antigens, which will likely be key in the propagation of an array of bNAbs capable of neutralizing multiple HIV-1 subtypes in each vaccine [143]. Lastly, researchers have found that implementing a self-amplifying sequence into mRNA designs, termed self-amplifying RNA (sa-mRNA), can further enhance the potency and duration of immunogenicity while potentially reducing the required dose concentration and cost [179,180]. 

Overall, mRNA vaccine technology has revolutionized the field of vaccinology, offering new hope for combating HIV-1 and other viral diseases. The rapid development, strong immune responses, adaptability, and reduced cost of mRNA vaccines make them a powerful tool in the fight against infectious diseases and beyond. Continued research and innovation will be essential to overcome existing challenges and fully realize the potential of mRNA vaccines.

### 4.4. Nanoparticle Vaccines

Nanoparticle vaccines utilize tiny particles, often made of proteins or lipids, to deliver an array of antigens or nucleic acids to the immune system. These particles can mimic the structure of viruses, enhancing the immune response by presenting genetic material in a way that resembles a natural infection [181,182,183]. Nanoparticles can be highly versatile, involving several unique forms including inorganic particles, lipid nanoparticles (LNPs), biopolymer particles, virus-like particles (VLP), and recombinant antigens [184]. Collectively, they have shown promise in improving vaccine efficacy and potency by markedly enhancing the immune responses. Nanoparticles can be engineered to display multiple copies of HIV-1 antigens, which can significantly boost the immune response. This multivalent presentation helps the immune system recognize and respond more effectively to the virus, by inducing a strong antibody and T cell responses. Moreover, nanoparticles offer a safe and effective alternative to live-attenuated viruses, which are considered too dangerous to use for an HIV-1 vaccine [185]. 

One of the major challenges in HIV-1 vaccine development is the virus’s high mutation rate. Nanoparticle vaccines can be designed to present conserved regions of the HIV-1 Env protein, which are less prone to mutation. This strategy aims to elicit bNAbs that can neutralize a wide range of HIV-1 strains. Nanoparticle engineering has shown promising results in several preclinical and clinical trials. A preclinical study utilized a single-component, self-assembling protein nanoparticle (1c-SApNP) conjugated to BG505 uncleaved prefusion-optimized (UFO) trimers to induce bNAb production in mice, rabbits, and NHPs. Moreover, these researchers reported that specific glycan modifications can further enhance the vaccine response [186]. In clinical studies, researchers designed an outer domain germline targeting version 8 (eOD-GT8) 60-mer nanoparticle capable of priming VRC01-class HIV-1-specific B cells for bNAb production [130]. More recently, this same group revealed that the eOD-GT5 60-mer induced a CD4 T cell response, demonstrating the highly effective immunogenic capabilities of this nanoparticle-based vaccine [132]. 

A major benefit of nanoparticle technology is that they can be combined with adjuvants—substances that enhance the body’s immune response to an antigen by stimulating the release of pro-inflammatory cytokines [187]. Recent research has shown that nanoparticle-based adjuvants can significantly improve the production of antibodies following HIV-1 vaccination. This includes a study which compared the immunogenicity of ConM SOSIP trimers as free proteins, conjugated to nanoparticles, and in combination with various adjuvants (squalene emulsion, MPLA liposomes, ISCOMATRIX, and GLA-LSSQ) to induce bNAb responses in rabbits [188]. Their findings indicated that ferritin nanoparticles not only enhanced the bNAb response but also elicited an even greater increase when delivered with GLA-LSQ adjuvant. These adjuvants have also been shown to help create a more robust and long-lasting immune response. For instance, the use of 3M-052, an imidazoquinoline adjuvant, when paired with PLGA nanoparticle-based vaccines was shown to induce sustained Tier 1 NAb production in NHPs [187,189]. While these studies highlight the efficacy of incorporating adjuvants into vaccination treatment [187], the use of these formulations have contributed to “vaccine hesitancy”, as side effects can include tissue inflammation, body aches, and fever in patients [190,191]. While their benefits significantly outweigh these potential side-effects, the use of adjuvants may be discouraged for patients with pre-existing autoimmune or inflammatory diseases. 

Nanoparticle technology is also being explored for vaccines against other viral diseases, including the flu. Traditional flu vaccines often have variable effectiveness due to the constantly changing nature of flu viruses. As mentioned, nanoparticle vaccines can be designed to present multiple flu antigens, potentially offering broader and more consistent protection. In 2022, researchers reported that an S60-HA1 pseudovirus nanoparticle displaying HA1 antigens induced strong antibody production in mice [192]. More recently, the Lei laboratory generated a mosaic influenza nanoparticle vaccine (FluMos-v1) which elegantly presents 2-4 recently identified HA strains [193]. FluMos-V1 is currently being tested in a clinical trial (NCT04896086), and if successful, will likely influence the design of nanoparticle vaccine design for many years to come [193]. In addition to the flu, Zika has also become a prime target for nanoparticle vaccine design. Preclinical studies have demonstrated that nanoparticle vaccines can induce strong immune responses against Zika in mice, and clinical trials are underway to test their efficacy in humans [194]. A third, and perhaps most prevalent target for nanoparticle vaccines is COVID-19. The pandemic dramatically accelerated the development of nanoparticle vaccines with several COVID-19 vaccines using LNPs to deliver mRNA or antigenic proteins showing high efficacy and safety. In a study involving NHPs, a ferritin-based, protein nanoparticle DCFHP with an aluminum adjuvant was capable of inducing bNAb production [195]. This success has highlighted the potential of nanoparticle technology for rapid vaccine development and deployment for other pathogens [196]. Finally, nanoparticle technology may be a viable candidate for RABV. Researchers have shown the Fagy-tagged glycoprotein domain III (G_DIII_)-ferritin nanoparticle conjugate to provide stable immunogenicity to female mice after a single dose [197]. 

Much like previously mentioned vaccine methods, nanoparticle technology for vaccine design comes with its own set of obstacles. Among these includes the difficulties in scaling up microfluidic technologies, which is known to be a common tool for LNP design and production [198,199]. In an effort to overcome this, researchers developed a unique silicon and glass microfluidic platform, which enables the manufacturing of mRNA-LNP vaccines to be dramatically increased [200]. Nanoparticles can also be sensitive to environmental conditions, requiring careful handling and storage. Developing formulations that enhance the stability of nanoparticle vaccines is crucial for their widespread use. Finally, while the use of LNPs can provide protection for mRNA from RNAse-mediated degradation, the nanoparticle formulation, particularly the excipient used, can markedly reduce LNP-mRNA stability [201]. Research assessing excipient/LNP-mRNA combinations to enhance shelf life is necessary for their global use. 

As with any new technology, nanoparticle vaccines must undergo rigorous testing to ensure their safety and efficacy. Navigating the regulatory landscape can be complex and time-consuming, but these findings surrounding nanoparticle-based vaccines make them a viable candidate to target HIV-1 and other diseases going forward.

### 4.5. Gene Therapy in HIV-1

Gene therapy, which involves the delivery of therapeutic genes (e.g. DNA, RNA) to cells or tissue, has made significant strides in cancer treatment [202,203,204,205,206,207]. Among the many different forms of gene therapies available, studies utilizing chimeric antigen receptor (CAR) T cell therapy to target cancer have showcased its tremendous efficacy [208,209,210]. This success has provided a robust framework for developing innovative CAR T-based HIV treatments, offering hope for more effective and potentially curative therapies [211,212]. CAR T cell therapy is a form of immunotherapy that modifies parental T cells to better recognize and attack specific targets. This process involves several key steps: (1) Collection: T cells are collected from the patient’s blood. (2) Modification: these T cells are genetically engineered in the laboratory to express CARs on their surface. CARs are synthetic receptors that can specifically recognize and bind to antigens on the surface of HIV-1-infected cells. (3) Expansion: the modified T cells are then expanded in a medium containing cytokines to promote their growth and create a large number of CAR T cells. (4) Infusion: the CAR T cells are infused back into the patient, where they seek out and destroy HIV-1-infected cells [213].

Among its many benefits, CAR T cell therapy for HIV-1 can be a highly versatile approach to HIV-1 vaccination. CAR T cells can be engineered to target the gp120 Env protein on the surface of HIV-1-infected cells. By designing CARs that recognize gp120, researchers aim to direct the immune system to specifically attack and eliminate HIV-1-infected cells [213,214,215,216]. Furthermore, recent advancements include the development of dual CAR T cells, which target two different epitopes on the HIV-1 gp120 protein. This dual targeting approach enhances the ability of CAR T cells to recognize and kill HIV-1-infected cells, reducing the likelihood of the virus escaping immune detection [217]. The success of CAR T cell therapy has been highlighted by the success of preclinical and clinical trials. Preclinical studies have demonstrated that CAR T cells can effectively suppress HIV-1 and eliminate HIV-1-expressing cells in vitro and in animal models. Notably, Anothony-Gonda et al found that anti-HIV-1 duo CAR T cell treatment resulted in a complete loss of HIV-1-infected PBMCs in mice spleens, the primary site of HIV-1-infection [218]. Due to this success, early-Phase clinical trials are now underway to evaluate the safety and efficacy of CAR T cell therapy in humans. A clinical trial at UC Davis is testing a novel CAR T cell therapy designed to control HIV-1 without the need for ART [218]. In 2024, Mao et al published findings from a Phase I clinical trial, demonstrating that molecularly engineered M10 CAR-T cells can reduce HIV-1 titers in PLWH [219]. 

While the aforementioned studies have highlighted the benefits of CAR T cell therapy for HIV-1 treatment, there are several challenges that this method will face going forward. First, the safety and tolerability of CAR T cell therapy is crucial. Early clinical trials are focused on evaluating potential side effects and determining the optimal dosing regimens. Another crucial goal going forward is to ensure that CAR T cells persist in the body long enough to provide the sustained control of HIV-1. Researchers are exploring ways to enhance the longevity and functionality of CAR T cells in vivo [220,221]. Finally, the cost and accessibility of CAR T therapy is something many need to consider. CAR T cell therapy is complex and expensive, requiring specialized facilities that may not be available in low-resource countries. Making this treatment accessible to a broader population will require significant advancements in manufacturing and cost reduction. Given its personalized nature, CAR T therapy is more acceptable for life-threatening illnesses like cancer. However, for HIV-1, it is crucial to consider whether this approach is practical for the millions of individuals living with the virus worldwide.

Another gene therapy approach that holds significant potential in the development of an HIV-1 cure is the gene editing technology CRISPR/Cas9. As this powerful tool can target and modify specific DNA sequences within host genomes, researchers began assessing whether CRISPR/Cas9 could be used to target the integrated proviral DNA within HIV-1 infected cells. Both Ebina et al and Hu et al demonstrated that the CRISPR/Cas9-based targeting of HIV-1 long terminal repeat (LTR) sequences in infected T cells could effectively block viral gene expression and replication [222,223]. Building on these findings, researchers have found that targeting multiple HIV-1 loci using two gRNAs can block replication while preventing viral escape [224]. Similar success has been shown by those utilizing CRISPR/Cas9 to target CCR5/CXCR4 expression [225,226] and reactivate latent HIV-1 reservoirs [227,228,229]. While these studies have highlighted the potential of CRISR/Cas9 technology in HIV-1/AIDS treatment, its efficacy in clinical trials has been delayed due to concerns over off-target effects caused by Cas9-mediated cleavage [230,231]. This warrants further research and new techniques to ensure safety for those receiving CRISPR/Cas-9-based HIV-1 therapeutics. 

### 4.6. Viral Vector-Based Expression of bNAbs

The concept of the viral vector-based expression of bNAbs emerged from the success of passive immunization with bNAbs to control HIV-1 replication [78,232,233,234,235,236,237]. Researchers recognized that delivering bNAbs to HIV-1 infected individuals could be an effective therapeutic approach. However, this approach relies on the shock-and-kill activation of latent virus and multiple bNAb infusions, making it costly and sometimes ineffective. To address these challenges, researchers developed recombinant adeno-associated viral (rAAV) vectors for the persistent expression of bNAbs and other HIV-1 inhibitors. This approach leverages gene therapy techniques, with rAAV delivering the genetic code for bNAbs into the body. The aim is for the rAAV to persist inside cells and act as a factory for producing bNAbs continuously, providing long-term protection against HIV-1. This method has shown promise in preclinical and clinical studies, demonstrating the potential to combat HIV-1 and develop into a viable vaccine strategy [238,239]. By harnessing the power of viral vectors, scientists are paving the way for innovative treatments that could revolutionize HIV-1 therapy and prevention.

The first study to use rAAV vectors in HIV-1 research was conducted in 2002, by Lewis et al. who expressed a b12 antibody targeting the CD4-binding site in mice, resulting in detectable b12 levels and HIV-1 neutralization. This approach was later adapted to co-express CD4-Ig and an SIV-inhibitor in monkeys, showing a promising response in some, but not all, animals. This early success led to numerous preclinical studies in the 2010s assessing the capabilities of rAAVs vectors, particularly in NHPs. Notably, Gardner et al generated rAAV vectors containing eCD4-Ig, an antibody-like protein composed of CD4 domains 1 and 2 fused to an IgG Fc with a sulfated coreceptor-mimetic peptide on the C-terminus of the Fc, which provided protection against SHIV-AD8 infection in monkeys. Another study by Seaman and colleagues confirmed the durable protection offered by AAV-expressed eCD4-Ig in rhesus macaques, emphasizing the potential of eCD4-Ig to provide long-term immunity against HIV-1 by continuously producing the protein within the host. The use of eCD4-Ig is particularly promising as it can bind to both the CD4 and coreceptor-binding sites of HIV-1 Env, significantly reducing the virus's fitness and ability to escape immune detection. Interestingly, eCD4-Ig exhibits exceptionally broad neutralization breadth compared to bNAbs, neutralizing all 270 HIV-1, HIV-2, and SIV isolates tested to date, each with 80% inhibitory concentration (IC80) values of less than 10 μg/mL [240]. rAAV delivered eCD4-Ig effectively protected rhesus macaques from SIVmac239. In 2015, another research group inoculated monkeys with rAAV expressing VRC07 bNAb, which remained detectable for up to 16 weeks and provided immunity to SHIV [241]. These studies underscore the potential of rAAV vectors to deliver eCD4-Ig, either alone or in combination with other bNAbs offering a promising approach for long-term HIV-1 protection and therapy. 

Despite these promising findings, to date, there has only been one clinical study completed utilizing this technique. This double-blind, Phase 1, randomized, placebo-controlled, dose-escalated trial assessed the immunogenicity of rAAV1 encoding bNAb PG9 (rAAV1-PG9DP). While PG9 was detected in serum and muscle biopsy, this was only in a small subset of volunteers [242]. These results, along with the detection of a T cell response, suggest the need for the further investigation and modification of this approach. The continued study of rAAV capabilities as a platform for an HIV-1 cure has been in parallel to its success in treating various other pathologies. Among these is its use to treat severe hemophilia B, which is a bleeding disorder characterized by individuals lacking factor 9 gene encoding for blood clotting factor IX protein. In a clinical trial, patients treated using rAAV showed detectable levels of factor IX in muscle tissue using various biochemical assays [243]. These findings demonstrate rAAV as a viable vector and approach in the development of an effective HIV-1 vaccine and cure. Currently, there is one clinical trial in progress (NTC03374202), which is assessing the rAAV8-mediated expression of bNAb VRC07 in PLWH [238]. 

There are several challenges the research community faces in pursuit of implementing rAAVs into HIV-1 therapeutics. The delivery of rAAVs can result in the immune system mounting an anti-drug antibody (ADA) response, which can target rAAVs and consequently reduce overall efficacy [244,245]. An ADA response can occur due to several factors, which includes individuals having preexisting Abs and immunity to rAAVs [246,247,248]. One approach being used to mitigate this issue is the packaging of AAV vectors with exosomes (exo-AAV) [249], which is a subclass of extracellular vesicles that contain a protective phospholipid bilayer [250]. Another solution is the testing and targeting of Abs to rAAV prior to treatment [251]. It is important to note that this solution also comes with its own set of challenges, as it requires specialized facilities and technical experience, therefore making it inaccessible to broad communities. ADA responses can also occur due to rAAV-encoded bNAbs being recognized as foreign to the host, resulting in an immune response-mediated reduction in rAAV efficacy [244]. To address these challenges, researchers are modifying lentiviral vectors and engineering pseudoviruses to selectively transduce splenic B-cells for the long-term expression of anti-HIV-1 antibodies without inducing an ADA response (Case no. 2023-237). B-cells can physiologically generate a wide variety of antibodies through gene recombination and mutation and develop tolerance to foreign proteins expressed in them. Thus, by using B-cells to express transgenes like anti-HIV antibodies, the immune system can be conditioned to accept these antibodies as part of the body, thereby avoiding an anti-drug antibody (ADA) response and enabling continuous, long-term antibody production without triggering immune reactions against them. Innovative work such as this holds promise for the functional curing and eradication of HIV-1 in the near future. 

### 4.7. Extracellular Vesicles 

As eluded to in the previous section, the use of extracellular vesicles in HIV-1 therapy and vaccine technology represents a research focus with tremendous potential on the horizon. Extracellular vesicles (EVs) are phospholipid membrane organelles released from cell surfaces, which are known for their ability to act as key signaling molecules in mediating cell-cell communication; this pivotal function is derived from their ability to protectively package and deliver biological material, including proteins, DNA, and RNA, to cells or tissues [252,253]. By virtue of these functional capabilities, researchers have begun assessing their ability to shock-and-kill HIV-1 reservoirs [254,255]. EVs can be targeted to specific HIV-1 infected CD4+ T cells, and through the packaging of key cargos, can activate these reservoirs [256,257]. Among the studies that have demonstrated this is Barclay et al, which showed that c-Src loaded EVs could trigger the PI3K/AKT-1/mTORE pathway, resulting in the reactivation of HIV-1 production in resting CD4+ T cells [257]. While there are no clinical trials to date utilizing EVs in HIV-1 therapeutics or vaccination, these studies have showcased their potential in this regard. Through exciting innovations in vaccine methodologies such as this, the research community maintains hope for a global HIV-1 vaccine in the near future.

## 5. Conclusions and Perspectives

In summary, advancements in HIV-1 vaccine and treatment strategies have shown significant promise in the fight against the virus. Continued research and clinical trials are essential to further advance the above-mentioned strategies and perhaps by integrating the novel techniques such as CAR T cell therapy and the targeted B cell transduction, engaging the cellular and humoral arm of the immune response, could enhance their efficacy and durability. Additionally, exploring combination strategies with other emerging technologies, such as CRISPR and mRNA vaccines, could further revolutionize HIV-1 treatment and prevention. However, one thing to keep in mind is that while mRNA HIV-1 Env-based vaccines can enhance antibody magnitude, traditional Env formats like gp120, gp160, and SOSIP have not successfully elicited bNAbs. Therefore, simply switching to an mRNA format is unlikely to improve outcomes. Innovative and carefully designed Env immunogens are essential to achieve the desired immune response.

The COVID-19 pandemic not only showcased the advancement of different technologies available for vaccine design, but also demonstrated the possibility to minimize the timeframe between clinical trial initiations to vaccine availability. To produce an effective SARS-CoV-2 vaccine, Phase 1-3 clinical trials were completed within a year. This rapid development of COVID-19 vaccines, achieved in less than a year, was unprecedented and involved global collaboration, significant funding, and accelerated regulatory processes. This was a remarkable achievement, as vaccine development typically takes several years, often spanning a decade or more. Moving forward, the methods used to enhance the rate of the recruitment and execution of COVID-19 clinical trials may elevate these processes for other pathogens including HIV-1. 

Thus, the future of HIV-1 research lies in the collaborative efforts of scientists, clinicians, and policymakers to translate these scientific breakthroughs into accessible and affordable treatments for all affected individuals. With sustained investment and innovation, the goal of an HIV-1-free world is within reach.

## Data Availability

No new data were created or analyzed in this study. Data sharing is not applicable to this article.
